# Proposing a framework for Health Impact Assessment in Iran

**DOI:** 10.1186/s12889-015-1698-1

**Published:** 2015-04-09

**Authors:** Ali Fakhri, Patrick Harris, Mohammadreza Maleki

**Affiliations:** Social Determinants of Health (SDH) Research Center, Kashan University of Medical Sciences, Kashan, Iran; Menzies Centre for Health Policy, School of Public Health, Sydney Medical School, the University of Sydney, Sydney, Australia; Department of Health Service Management, School of Health Management and Information Sciences, Iran University of Medical Sciences, Tehran, Iran

**Keywords:** Health impact assessment, Healthy public policy, Health policy

## Abstract

**Background:**

Health impact assessments (HIA) of policies and projects are conducted differently in different contexts although there has been less HIA research to date in non-western countries. Global HIA research has however suggested that the technical conduct of HIAs is tied to broader conditions and influences to do with decision making and policy development.

This study was conducted to develop a conceptual framework for progressing HIA in Iran including all factors influencing HIA planning and practice.

**Methods:**

A comprehensive review of the international HIA literature identified core characteristics and principles. Then key informant interviews (n = 14) identified Iranian perspectives about factors influencing HIAs practice. These two stages resulted in a conceptual framework for HIA planning and practice including influencing factors and HIA content that was confirmed by our participants using e-Delphi technique.

**Results:**

91 HIA characteristics were organized into 20 categories. The interviews showed that four core concepts i.e. context, actors, HIA principles and policies and HIA capacities influence HIA practice in Iran. Comprehensive content of HIA considering all health dimensions and health determinants, assessing health inequalities, appropriate HIA type, quantification and participation is formed under influence of the above mentioned four factors. The study also demonstrated need to redefine the HIA principles and make decision about integration of HIA in Environmental Impact Assessment and also about the level of HIA before implementing HIA. The e-Delphi resulted in expert consensus on the variables, concepts, and their relations in proposed framework.

**Conclusions:**

Progressing HIA practice in Iran is perceived locally as subject to similar contextual conditions to those identified in the international literature. Further we have demonstrated the utility of mixed methods to progress HIA implementation in differing country contexts.

## Background

Health system authorities not only seek ways to provide the best health care but also improve the health of populations by considering the wider determinants of health [1,2]. One established way of doing this is the use of Health Impact Assessment (HIA) to aid decision makers by predicting positive and negative health impacts of proposed policies, programs and projects. However, the reality of influencing policy [3] and project [4] decisions is that these involve a complex set of factors. Considering this broad range of factors is, therefore, essential for the planning and effective conduct of HIA [5-7].

This placing of HIA within the array of influences on decision-making suggests that HIAs are conducted differently in different contexts. There has been empirical investigation into these factors in Europe [6], the U.S. [8] and globally [7,9,10]. Developing countries have started to implement HIA. For example, Thailand health system has developed HIA by conducting HIA case studies, developing HIA guideline and introducing HIA rules and procedures document [11-13]. Nonetheless, there has been no research to date which has focused on how adaptable HIA is in Iranian context. However each country requires its own policy frameworks and procedures for HIA that are adapted to the structure and legislation of local ministries and to its environment and communities [14].

In Iran, environmental impact assessment started in1990 but health had not been detailed in EIAs with only some projects i.e. industrial estates, wastewater treatment and some impacts i.e. air pollution have been assessed by academics using EHIA guidelines. The ignoring the health in EIAs and also an increased emphasis on the social determinants of health, caused the Fifth Economic, Social and Cultural Development Plan (2010–2015) [15] oblige the Ministry of Health and Medical Education (MoHME) to define national standards for ‘Health Annex of Developmental Plans’ aimed to implement HIA. This means HIA would be a regulatory requirement in Iran. This impetus provided the opportunity to investigate the perspectives of experienced Iranian practitioners and policy makers about the applicability of globally defined factors to progress a comprehensive HIA practice in Iran.

## Methods

A comprehensive review of the international HIA literature identified core characteristics of HIA. A qualitative study identified Iranian perspectives about factors influencing HIAs practice. These two stages resulted in a conceptual framework for HIA implementation and practice confirmed using e-Delphi technique. Ethical approval was provided by the Complementary Educations Council of the School of Health Management and Information Sciences, Iran University of Medical Sciences on February 2012.

### Data collection

This study was informed by two data sources. We first undertook a literature review to identify globally defined HIA characteristics and wider influences on HIA practice. Stakeholder interviews where then undertaken to understand local contextual issues and factors influencing HIA. We used the findings from both of these stages to propose a conceptual framework.

#### Literature review; Identifying HIA characteristics

We extracted HIA characteristics from a thesis in Persian [16] previously categorized and published as underlying principles for Health Impact Assessment [17]. According to primary source, the phrase “Health Impact Assessment” was searched for in Pubmed and Scopus databases from 1995 to 2012 resulted in 322 and 660 results respectively while 278 were common. The review was not systematic. Reviewing titles and abstracts in the peer reviewed literature, papers were excluded if they concerned or reported specific cases of HIAs. This review resulted in a total of 201 papers with background knowledge about HIA. Papers were included if they written in English and dealt with generic characteristics of HIA. Aim to data saturation. we started the review with common results by this order; written by authors who have the more papers in the Web of Sciences in this subject; extracted from international workshops and published by World Health Organization. Review was stopped by 63 papers reaching data saturation. For example, some papers referred to HIA characteristics and principles as the terms of characteristics [5,18], principles [19], requirement [20], prerequisites [21], key topics [22], key tasks [23], HIAs main value [24], criteria and parameters [25], broad factors [26] and other papers described one or more characteristic and principle without mentioning a specific term.

#### Key informants Interviews; Identifying contextual factors

A purposive sample of participants was selected for their experience with HIA or related areas. Unstructured interviews, which took from 30 to 90 minutes, were conducted until data saturation was reached (n = 14). Participants’ characteristics were: four members of Health Annex National Standards Committee responsible for developing National standards for HIA, three from Environmental Protection Organization, three from the Social Determinants of Health Secretariat in MoHME, and four Academic experienced in Environmental Health Impact Assessment and HIA have conducted their HIA projects as an academic activity in different field e.g. industrial estates documented in Persian. Interviewees were informed about interview and they signed a consent form before interview. AF conducted the interviews which focused on identifying HIA characteristics and principles and their influencing factors. Interviews were tape-recorded. The interviews revolved around two broad questions;

1. What are the factors influencing health impact assessment in Iran?
2. What are the dimensions of doing a comprehensive health impact assessment which need to be considered?

#### E-Delphi consensus; confirming the framework

Our participants in the interviews were our experts to reach consensus using Delphi method. Proposed framework was e-mailed to participants (n = 14). These data were collected over 1 month from December 2013 to January 2014. Of 14 experts in HIA and related fields from previous stage of the study, 14 completed Round 1 and 13 completed Round 2. Percent agreement was computed to establish consensus.

### Data analysis

Using MS word to conduct a conventional content analysis of the included literature, AF and MM coded the identified HIA characteristics and principles until data saturation occurred. Codes were categorized based on their similarities. For example the term of “political environment” was used to cover, “political context”, “political environment” and “political commitment”.

Using thematic content analysis, AF and MM coded transcribed texts until the data were saturated to identify Iranian contextualized perspectives concerning factors influencing HIA and about the dimensions of doing a comprehensive HIA. For example participants emphasized the role of economic conditions influencing HIAs practice, which we therefore developed as a parameter influencing the HIA content.

**Figure 1:**
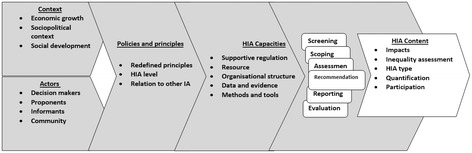
**HIA conceptual framework.**

In the following way we coded these parameters and categorized them for developing the conceptual framework. From the literature we categorized 91 codes and 24 categories (see Table 1). Then, using the interview data, we further reduced these categories into 20 contextualized parameters, five subthemes and two themes (see Table 2). Finally, the conceptual framework was formed considering emerged relations between five concepts from the literature and interview data that was validated by our participants using e-Delphi technique 2 Round to establish consensus (see Figure 1).

## Results

### The HIA characteristic and principles in literature

Considering 171 codes identified from the literature review [16], by deleting identical concepts, 91 codes (HIA characteristics) were developed which were subsequently categorized into 24 categories (Table 1).

**Table 1:** **The HIA characteristics extracted from literature review and their categorizations**

**The characteristics of HIA in the papers**	**Categories**
Range of impacts considered	**- Health impacts **(Health dimensions and health determinants)
Focus of HIA	
Multidisciplinary	
Comprehensive	
Potential impacts	
Health determinants	
Type of HIA (rapid, intermediate, comprehensive)	**- Type of HIA** (rapid, intermediate, comprehensive)
Effective stakeholder participation	**- Participation**
Cross-sectoral approach	
Multidisciplinary	
Intersectoral engagement	
Health advocacy	
Quantification	**- Quantification**
Consideration of health inequalities	**- Inequality assessment**
Supportive institutional context	**- Economic conditions**
Politicised context	**- Political environment**
Political commitment	**- Social context**
Clear bureaucratic mandate	
Politico-administrative environment	
Policy process	
Community context	
Context for HIA use	
Governance	
Social acceptance	
Socio-political environment	
Policy framework and procedures	
Socio-political context	
Developed or developing economy	
Population attitude and belief	**- Community**
Stakeholder position	**- Key informants**
Engaging a diverse set of stakeholders	**- Project proponents**
Engagement with impacted communities	**- Decision makers**
Community and stakeholder involvement	**- Assessors**
Policy makers involvement	
Community participation	
Participation in HIA	
Shared ownership	
Base	**- HIA principles**
General stream	**- HIA values**
Principles and values	**- Relation to other IAs**
Timing (retrospective, concurrent, prospective)	**- Level of HIA**
Influence decision making	
Predictive	
Sector of intervention	
Steering group	
Who conduct HIA	
Conceptual framework	
Robust and broad theoretical foundation	
Model of health	
Using of structured framework	
Systematic approach	
Structured and stepwise process	
Level of HIA	
Administrative level of conducting HIA	
Relation to other forms of IA	
Integration with economic appraisal	
Institutionalization	**- Statutory requirements**
Statutory requirements	
Clearness of responsibility and Accountability	
Voluntary vs. Regulator	
Funding	**- HIA resources**
Resource needs and limitations	
Economic feasibility	
Cost-effectiveness of HIA	
Skilled facilitation	**- Human capacities**
Competent practitioners	**- Organisational capacities**
Capacity for professional development	
Capacity and workforce needs	
Capacity-building mechanisms	
Expertism	
HIA capacity building	
Administrative frameworks	
Institutional infrastructure	
Technical appropriateness	**- HIA methods & tools**
End-user–friendly implementation	
Guidance for health analysis	
Tools and methods	
How to Identify health impacts	
Methods and techniques	
Uncertainty	
Availability of data	**- Appropriate data**
Value of evidence	**- Appropriate evidence**
International HIA experience	
Published literature consideration	
Appropriate data sets	
Evidence base HIA	
Explicit source of evidence	
Type of data	
Examples and case study	
Use of appropriate evidence	
Robust evidence	

### Interview findings

In the qualitative interviews (translated here from Persian to English) participants mostly referred to the broad conditions influencing HIA practice. Participants referred to the different parameters influencing HIA’s role in decision making. Interviewees believed that because Iran is now focused on economic growth this necessitates considering the environment and health more than before. One participant explained;

“….in countries with rapid economic growth, something is sacrificed for other things, for instance the environment and health are sacrificed for economic development…” (p1)

Participants were concerned about challenges in HIAs development being perceived to prolong developmental planning which could be out of step with political imperatives and the economic motivations of development proponents. For example one participant engaged in Policy Making Council of MoHME said:

“…Election promises means they [elected politicians] are under pressure and don’t acquiesce for projects to be slowed or stopped. Authorities’ attitude is a major challenge…” (p7)

However, participants also felt that HIAs focusing on broad concerns to society as opposed to the interests of individuals was an enabler for HIA success in Iran. One participant explained;

“…Social development which emphasizes the subordination of individual interests to general interests causes the social acceptance of HIAs recommendations …” (p4)

Additionally, participants believed that although principles and standards for HIA practice have been developed internationally (i.e. the Gothenburg consensus), there is a need to define HIA principles which consider Iran’s cultural and socio-political context.

All participants identified that supportive legislation, financing, skilled human resources and organizational capacity, appropriate data and tools are prerequisites for effective and successful HIA. They felt that all these are prerequisites, or ‘HIA capacities’, that must be built before HIA implementation.

“…we have good capacity in specialist human resources [for HIA] but they also should be trained and skilled…” (p6)

Some believed that HIA practice must be integrated with Environmental Impact Assessments of projects. There was however some consensus that these issues of integration vs. stand alone HIA be resolved prior to the development of an HIA system in Iran. Similarly participants also felt that decisions about conducting HIAs at a policy level in addition to projects should be made before the development of an HIA system.

In terms of the content of HIAs, interestingly the importance of incorporating indirect health dimensions when assessing impacts was regarded by one participant;

“…calculating mortality isn’t enough. We must take into account stress of workers and their families in primary stages of a developmental project that is built in another province…” (p4)

Also in terms of HIAs content some participants believed that direct community participation is not possible, with others suggesting that because of potential dissension in communities HIAs should avoid involving them and instead look to involving community representatives.

‘….If we do not involve the community, HIA purpose will not be achieved but we cannot involve the community directly. We have to involve the formal representatives of the population…’ (p3)

Different beliefs were also detected about the use of data in HIA. Conducting rapid HIAs, which involve less data, was seen as acceptable at the policy level but not in the assessment of projects:

‘…rapid assessment is a way to avoid of data deficit… It is acceptable for health impact assessment of policies and laws that time is restricted but it is not enough for assessing of projects…’ (p1)

Although where possible data quantification was seen as needed;

‘…decision makers like numbers and statistics. I think quantification is needed although I agree a few prerequisites are necessary for this…’ (p7)

Finally data was discussed in relation to health inequalities,

‘…I think if we conduct HIA, we will able to decrease health inequalities… We have to gather the data about all subgroups if the money permits…’ (p11)

### The developed conceptual framework

Five core factors emerged from the literature and interviews; four as influencing factors and one as how to do HIA. The term of “HIA Context” was used to cover all contextual parameters. “HIA Actors” was selected to cover the all stakeholder’s attitude. “HIA Principles and Policies” covered the accepted core principles and adopted policies concerning HIA. “HIA Capacities” referred to any prerequisites for conducting HIA. Finally “HIA Content” referred to HIA characteristics that detail the ‘doing’ of comprehensive HIAs during defined process. These factors considering their role in HIA as influencing factor or HIA content based on the literature and participants’ viewpoints formed the basis of the conceptual framework (Table 2). Figure 1 shows the proposed conceptual framework diagram. The framework was confirmed by our participants during two Rounds by e-Delphi method coming to 80% agreement on parameters, concepts and their relations [27].

**Table 2:** **Contextualized parameters and supposed causal influencing factors identified from the interviews and literature**

**Contextualized parameters**	**Supposed causal influencing factors**	**Contextualized parameters**	**Different HIA practices**
Economic growth	Context	Health impacts	HIA content
Socio-political development		HIA type	
Social development		Health inequalities assessment	
Policy and decision makers	Actors	Quantification	
Key informants		Participation	
Community			
Proposal proponents			
HIA principles	Redefined principles and policies		
HIA level			
Integration to EIA			
Supportive regulations	Capacities		
Resources			
Organizational structure			
Methods and tools			
Data and evidence			

## Discussion

This study has proposed a framework for including HIA in policy making, planning and project development in Iran. By considering the range of factors involved in progressing HIA, the developed framework incorporates the broad parameters which influence HIA’s content and practice.

The results show that globally defined HIA characteristics and principles are largely supported in the Iranian context. At the same time this is the first study to develop a framework of how these various factors are perceived to play out in the application of HIA, in this instance in Iran. This provides local, non-western, contextual support for global research showing that HIA practice requires paying attention both to the external tactical or broader institutional factors as well as the technical issues related to HIAs conduct and practice [7]. For example, in Iran the results emphasize the broad influence of economic conditions, where the focus on economic growth and development influence the perceived socio-political usefulness of HIA and whether sufficient funds can be allocated to the practice of HIAs [6,28]. In the public policy arena the broad context in which HIAs are undertaken have been shown to be institutional, structures (the entities and rules within organizations or systems which influence policy making), actors (the stakeholders involved in policy making) and ideas (the content of policy making) as factors which work to create policy change [29]. We have similarly demonstrated that in Iran the ‘context’, which is largely structural, ‘actors’, which concern the people involved in progressing HIA, and ‘principles’, which largely concern the ideas which HIA has to offer, are each significant in progressing HIA as a technical process to influence decision-making. Our results support the need, in Iran, for legal commitments, financial and human resources and the development of supportive institutions [6,10] as well as technical requirements also in terms of methods, appropriate data and evidence [30,31] to influence decision making. We have also provided empirical support that these enablers have been also recognized as required to progress HIA in non-western countries [14].

However, our results also suggest that more work is required to understand the role of actors in the Iranian context. Contrary to expectation, viewpoints of some participants from Iran in this study concerning the role of actors i.e. policy and decision makers and proposal proponents were not positive. However, they suggested that actors do play an essential role. More detailed research is required to confirm the role of actors in progressing HIA in Iran.

The main purpose of this study was to qualitatively identify the various factors required to progress HIA in Iran. We suggest however that the results here are seen as initial and time limited. HIA has not progressed in Iran at the same level as in other countries, for example in Europe [6], the U.S. [32] or Australia [33]. In future more detailed quantitative work can confirm and refine our proposed framework both in Iran and in other contexts and countries response to call to develop an international HIA consensus that moves the field forward [34].

## Conclusions

This is a study which has developed categories to navigate the practice of HIA as a decision making tool. The present approach to HIA in Iran is an environmental health approach concerning environmental determinants of projects. Our framework suggests HIA could be modified to become a healthy public policy approach that considers all social determinants of health at multiple levels: project, programs, plans and policies. So in Iran it is necessary to make decisions about conducting of HIA for programs and policies and redefining of HIA principles before the implementation of an HIA system. It is also needed to make the decision about integrating of the HIA to Environmental Impact Assessment before implementation rather during the assessment. We have shown that experienced practitioners and policy makers in Iran feel that the most important aspects about HIA are context, redefined principles and HIA capacities. There are complex inter-relations between factors influencing HIA role on decision making. We have proposed an important conceptual framework for planning, progressing and conducting HIA in different contexts.
